# Correction: Multi-agent systems powered by large language models: applications in swarm intelligence

**DOI:** 10.3389/frai.2025.1771737

**Published:** 2026-01-22

**Authors:** Cristian Jimenez-Romero, Alper Yegenoglu, Christian Blum

**Affiliations:** 1 ETIS Laboratory, ENSEA, CNRS, UMR8051, CY Cergy-Paris University, Cergy, France; 2 Independent Researcher, Jülich, Germany; 3 Artificial Intelligence Research Institute (IIIA-CSIC), Bellaterra, Spain

**Keywords:** agent-based modeling, large language models, LLM-guided agents, simulation, swarm intelligence

Wilensky, U. (1999). *NetLogo*. Evanston, IL: Center for Connected Learning and Computer-Based Modeling, Northwestern University. Available online at: http://ccl.northwestern.edu/netlogo/ (Accessed December 18, 2025) was not cited in the article. The citation has now been inserted in **Introduction**, ***1.2*** ***Motivation***, Paragraph number 2 and should read:

The motivation and contribution of this work are found in the presentation of a toolchain that integrates LLMs with agent-based simulations within the NetLogo environment (Wilensky, 1999, Tisue and Wilensky, 2004; Amblard et al., 2015), a platform widely recognized in the complexity science community for its robustness and versatility.

Wilensky, U. (1997). *NetLogo Ants Model*. Evanston, IL: Center for Connected Learning and Computer-Based Modeling, Northwestern University. Available online at: http://ccl.northwestern.edu/netlogo/models/Ants (Accessed December 18, 2025) was not cited in the article. The citation has now been inserted in **3 Experiment 1: ant colony foraging simulation** and should read: As mentioned above, this experiment is based on the ant foraging model implemented in the NetLogo library (Wilensky, 1997, see https://ccl.northwestern.edu/netlogo/models/Ants).

Wilensky, U. (1998). *NetLogo Flocking Model*. Evanston, IL: Center for Connected Learning and Computer-Based Modeling, Northwestern University. Available online at: http://ccl.northwestern.edu/netlogo/models/Flocking (Accessed December 18, 2025) was not cited in the article. The citation has now been inserted in section **4 Experiment 2: bird flocking simulation** and should read: As mentioned before, the bird flocking model of NetLogo (Wilensky, 1998, see https://ccl.northwestern.edu/netlogo/models/Flocking) is an implementation of the famous Boids model from Reynolds (1987).

The original version of this article has been updated.

